# Identification of Anti-Collapsin Response Mediator Protein 2 Antibodies in Patients With Encephalitis or Encephalomyelitis

**DOI:** 10.3389/fimmu.2022.854445

**Published:** 2022-04-11

**Authors:** Kaibiao Xu, Dongmei Wang, Yan He, Shengnan Wang, Guanghui Liu, Yue Pan, Haishan Jiang, Yu Peng, Fenliang Xiao, Yihua Huang, Qiqi Wang, Yongming Wu, Suyue Pan, Yafang Hu

**Affiliations:** Department of Neurology, Nanfang Hospital, Southern Medical University, Guangzhou, China

**Keywords:** neurologic autoimmune diseases, brain inflammation, inflammatory encephalomyelitis, CRMP2 protein, autoantibodies

## Abstract

**Background and Purpose:**

An increasing number of autoimmune encephalitis (AE)-associated autoantibodies have been successfully characterized. However, many cases of AE remain unexplained on account of unknown antibodies. The aim of the present study was to identify a novel antibody against collapsin response mediator protein 2 (CRMP2) in suspected AE patients.

**Methods:**

A patient’s serum and cerebrospinal fluid samples tested negative for known AE antibodies; however, strong immunolabel signals were observed in the neuronal cytoplasm of the cortex, hippocampus, and Purkinje cells on rat brain sections. Immunoprecipitation from the rat brain protein lysate, followed by mass spectrometry analysis, was used to identify the targeting antigen. Western blotting and cell-based assay with antigen-overexpressing HEK293T cells were used for antibody specificity, epitope, IgG subtype determination, and retrospective study.

**Results:**

An antibody against CRMP2, a synaptic protein involved in axon guidance, was identified. The immunostains of the patient’s samples on rat brain sections were eliminated by pre-absorption with HEK293T cells overexpressing CRMP2. The samples specifically immunoreacted with CRMP2, but not with CRMP1, CRMP3, CRMP4, and CRMP5. The C-terminus of CRMP2 with 536 amino acids contained the epitope for antibody binding. The subtype analysis showed that the anti-CRMP2 antibody was IgG4. Furthermore, a screening of 46 patients with neurological disoders and neuro-cytoplasm immunostainings on rat brain sections resulted in the identification of anti-CRMP2 antibodies in a case of encephalomyelitis. The two patients responded well to immunotherapies.

**Conclusions:**

This study discovered that a novel anti-CRMP2 antibody was associated with suspected AE and thus should be included in the testing list for AE.

## Introduction

Autoimmune encephalitis (AE) encompasses a large category of inflammatory disorders mediated by immune responses against neuronal intracellular antigens, cell surface, or synaptic antigens, which, in some cases may be accompanied by neoplasia. Intracellular antigens include Hu (anti-neuronal nuclear antibody type 1, ANNA1), Ri (ANNA2), and Ma2 in the nucleus and Yo (Purkinje cell cytoplasmic antibody type 1, PCA1), amphiphysin, glutamate decarboxylase 65-kDa isoform (GAD65), and Kelch-like protein 11 (KLHL11) in the cytosol, which cause a T cell-mediated immune response. As it has been previously reported, some of these cases respond to immunotherapy (1–4). Most AEs that are caused by autoantibodies (auto-Abs) against antigens on the neuronal surface or synaptic proteins, such as N-methyl-D-aspartate receptor (NMDAR), leucine-rich gliomainactivated 1 (LGI1), contactin-associated protein 2 (Caspr2), gamma-aminobutyric acid (GABA) receptors (A/B), alpha-amino-3-hydroxy-5-methylisoxazole-4-propionic acid (AMPA) receptors, dipeptidyl-peptidase-like protein-6 (DPPX), delta/notch-like epidermal growth factor-related receptor (DNER), dopamine-2 receptor (D2R), metabotropic glutamate receptor 5 (mGluR5), voltage-gated calcium channel alpha-2/delta subunit (Ca_V_α2δ), and glutamate kainate receptor subunit 2 (GluK2), respond well to immunotherapy (1, 5–9). Thus, intensive testing of these antibodies (Abs) in suspected AE patients has an important role in guiding the diagnosis and treatment of the disease. Despite this, the cause of many cases of AE remains unexplained because of a limited number of known Abs (10–12). Consequently, identifying additional Abs is urgently needed.

The family of collapsin response mediator proteins (CRMPs) comprises five homologous members, *i*.*e*., CRMP1–5, which are cytosolic microtubule-associated phosphoproteins and have important roles in dendrite and axonal guidance, regulating migration and synaptic dynamics (13). Structurally, CRMP1–4 share 69–76% amino acid (aa) identity, while they are less similar to CRMP5, with which they share identity of approximately 50% (14). CRMPs are highly expressed in neurons in the cortex, hippocampus, cerebellum, immature, and mature oligodendrocytes (15). Auto-Abs against CRMPs lead to neurological disorders involving symptoms and signs related to central and peripheral neuron systems. Abs against CRMPs, except CRMP2, have been reported in several cases of encephalitis. Anti-CV2/CRMP5 Ab, one of the well-characterized paraneoplastic neurologic syndrome antibody, is frequently associated with encephalomyelitis and sensory neuronopathy. Polyradiculoneuropathy, retinopathy, myelopathy, limbic encephalitis, and cerebellar ataxia are common neurologic accompaniments (16). Previously, eight cases of anti-CV2/CRMP5 sera from patients with autoimmune myelopathy were found to be anti-CRMP3 Ab-positive, where one case also had anti-CRMP1 Ab, while the other had anti-CRMP1, 4 Abs (17). Abs to CRMP3,4, coexisting with GAD65, were found in a case with subacute limbic encephalitis and thymoma (18).

CRMPs Abs have also been found in other disorders, *e*.*g*., anti-CRMP1/2 Abs have been reported in autoimmune retinopathy patients (19). A previous study revealed that Abs to CRMP1/2 in maternal sera increased the occurrence of autism spectrum disorders in children (20). The presence of Abs to CRMP2 and/or GFAP in the acute phase of spinal cord injury increased the occurrence of neuropathic pain 6 months later (21). Herein we presented a case of suspected encephalitis with an identified anti-CRMP2 antibody. To elucidate the clinical features of patients with anti-CRMP2 antibody, a retrospective study with subjects of neurological disorders was conducted, revealing another patient with a positive anti-CRMP2 antibody.

## Materials and Methods

### Case Presentation

A 38-year-old female (P1) patient was admitted due to persistent dizziness, visual rotation, recurrent nausea and vomiting, and fever that lasted for 8 days. The patient also reported feeling unstable while walking and was suffering from slurred speech for 1 day. The fever was mild, and the highest body temperature was 38.5°C. On the first day, some medications were given by a local clinic to relieve the vomiting and fever. Three days later, the dizziness worsened and was accompanied by recurrent nausea and vomiting that started after the patient underwent a root canal treatment for chronic pulpitis. The patient was admitted to a local hospital on the next day. The cranial computed tomography (CT), magnetic resonance image (MRI), and magnetic resonance angiography (MRA) results were normal. Anti-platelet treatment and antibiotics were given to treat the dizziness and fever. However, the symptoms deteriorated with slurred speech and difficulty walking. The patient’s past medical history was not remarkable, except for glucose-6-phosphate dehydrogenase deficiency without symptoms. The patient was allergic to many products, including shrimp and albumin. On admission to our center, the physical examinations revealed signs of cerebellum damage, including scanning speech, nystagmus, and bilateral finger–nose test instability. Slight neck stiffness was noticed.

The CSF examination revealed a normal opening pressure of 148 mmH_2_O with only a slightly elevated white blood cell count of 38 cells/µl (90% monocytes). The CSF glucose (3.69 mmol/l, normal range: 2.80–4.50 mmol/l) and protein (0.43 g/l, normal range: 0.15–0.45 g/l) were within the normal range. The results of screenings of paraneoplastic antigens and serological autoantibodies, including anti-nuclear, anti-thyroid peroxidase, and anti-thyroglobulin Abs, were all negative. The serum anti-*Mycoplasma pneumoniae* (*M.P.*) IgM tests resulted positive two times within the first week after admission and weakly positive at 2 weeks later. The serum anti-Widal’s O test was positive with a titer of 1:80 on admission and conversed to negative at 2 weeks later. The next-generation sequencing (Vision Medicals, Guangzhou, China) of CSF sample detected 64 unique reads against *M.P*. The CSF test was negative for AE Abs, but positive by tissue-based assay (TBA).

**Figure 1:**
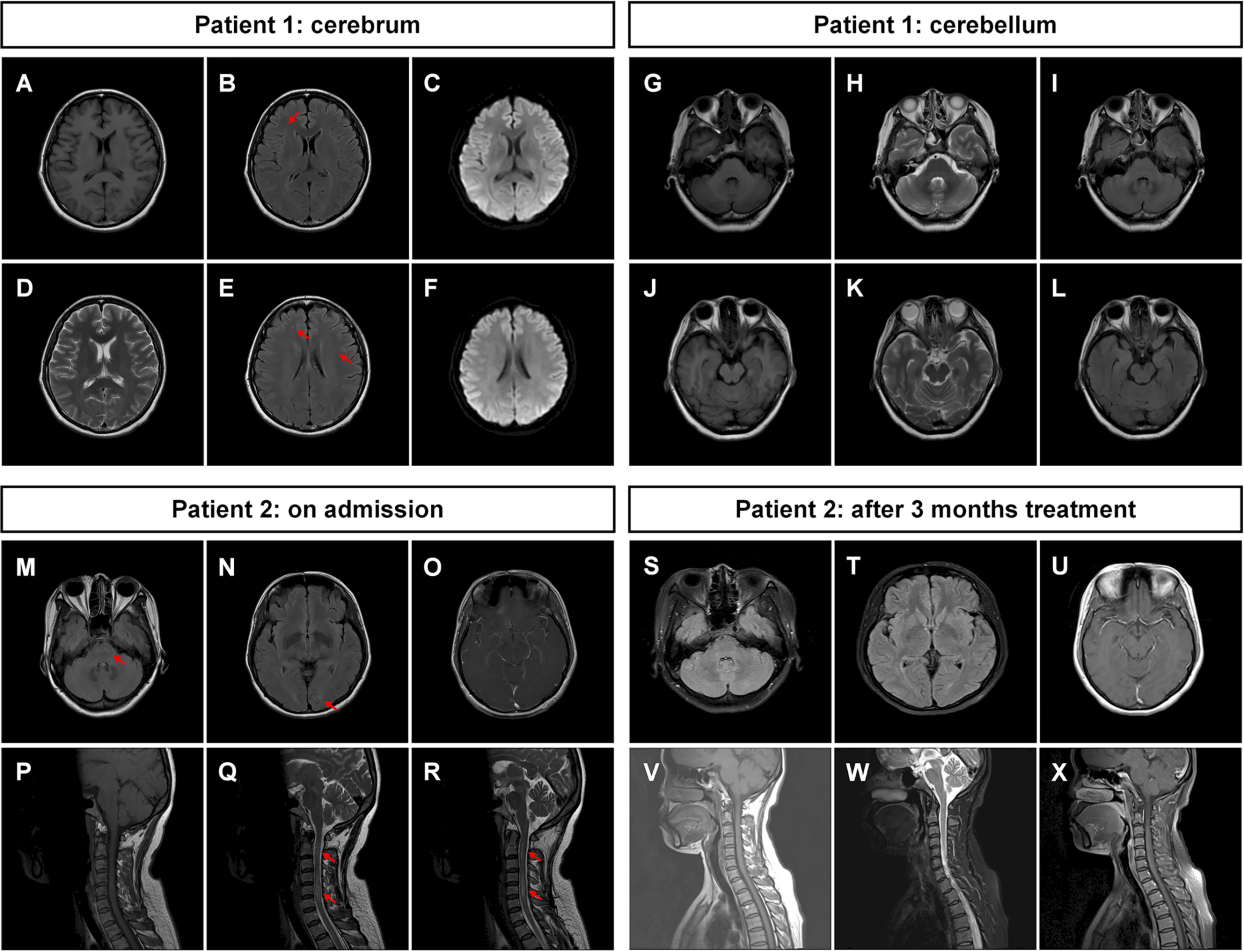
Magnetic resonance images of the patients. **(A–F)** Brain MRI of patient 1 was not remarkable with mild whiter matter abnormalities (indicated by arrows). **(G–L)** The MRI of cerebellum was not remarkable. **(M–O)** Brain MRI of patient 2 showed multiple abnormal signals in white matter in the bilateral cerebral hemisphere and brainstem (indicated by arrows). **(P–R)** The spine MRI of patient 2 showed long-segment spinal cord lesions from medulla to the C6 segment (indicated by arrows). After 3 months of treatment, **(S–U)** the brain MRI of patient 2 showed that the abnormal signals in the white matter and brainstem were reduced compared with those in **(M–O)**. **(V–X)** The cervical MRI showed that the original abnormal signals in the spinal cord had disappeared. T1-weighted images: **(A)**, **(G)**, **(J)**, **(P)**, **(V)**; T2-weighted images: **(D)**, **(H)**, **(K)**, **(Q)**, **(R)**, **(W)**; T2 fluid-attenuated inversion recovery sequence: **(B)**, **(E)**, **(I)**, **(L)**, **(M)**, **(N)**, **(S)**, **(T)**; T1-weighted images with contrast: **(O)**, **(U)**, **(X)**; diffusion-weighted imaging sequence: **(C)**, **(F)**.

The patient’s symptoms deteriorated during hospitalization, and she was experiencing recurrent nausea and vomiting. The patient also became apathetic. The results of the physical examination showed opsoclonus–myoclonus involving the head and both arms. It was diagnosed with intracranial *M.P.* infection and possible secondary immune-mediated encephalitis. Azithromycin, doxycycline, intravenous methylprednisone (MP, 40 mg/day), and intravenous immunoglobin (IVIG, 0.4 g/kg/day) were prescribed. Levodopa and clonazepam were also given to control the myoclonus. The treatment successfully relieved the mild dizziness and opsoclonus. The brain MRI was repeated, and the result was not remarkable with mild white matter degeneration (**Figures 1A–L**). The patient was discharged to a local hospital for rehabilitation. During the follow-up study, the patient partially recovered with dizziness and had a modified Rankin Scale (mRS) score of 2 at 3 months (**Table 1**).

**Table 1:** Clinical features of the patients with anti-CRMP2 antibodies.

Parameters	Patient 1	Patient 2
Gender	Female	Female
Age (year)	38	42
Symptoms	Dizziness, fever, slurred speech, visual rotation, unstable walking, opsoclonus–myoclonus	Headache, nausea, and vomiting, left chest pain, right arm fatigue, numbness in both legs
CSF	WBC: 38 cells/μl (90% monocytes), glucose: 3.69 mmol/l^a^, protein: 0.43 g/l	WBC: 240 cells/μl (90% monocytes), glucose: 2.64 mmol/l, protein: 0.80 g/l
Autoimmune Abs	Negative:	Negative:
	AE Abs^b^ (CSF and serum), anti-CRMP5 Ab (serum), anti-Homer3 Ab (serum), thyroid-related Abs^c^ (serum), remaining serum autoimmune Abs^d^	AE Abs (CSF and serum), anti-AQP4 Ab (CSF and serum), anti-MOG Ab (CSF and serum), anti-GFAP Ab (CSF), thyroid related Abs (serum), remaining serum autoimmune Abs^e^
	Positive: N/A	Positive:
		Anti-histone Ab (serum)
TBA	Serum (+), CSF (+)	Serum (+), CSF (-)
Infection tests	Serum anti-*M.P.* IgM Ab (+), CSF NGS: *M.P.* (+)	T-spot (-), TB DNA (-), X-Pert (-), AFB (-)
MRI	Not remarkable, mild white matter abnormalities	Multiple abnormal signals in white matter in bilateral cerebral hemisphere and brainstem, long-segment spinal cord lesions from medulla to C6 segment
Tumor	Tumor antigens/biomarkers^f^ (-)	N/A
Diagnosis	Encephalitis	Encephalomyelitis
Immunotherapy	IVIG, MP	MP
Prognosis (3 months mRS)	2	0

Abs, antibodies; AE, autoimmune encephalitis; AFB, acid-fast bacillus test; AQP4, aquaporin 4; CSF, cerebrospinal fluid; CRMP5, collapsin response mediator protein 5; GFAP, glial fibrillary acidic protein; Homer 3, homer scaffold protein 3; IVIG, intravenous immunoglobin; MOG, myelin oligodendrocyte glycoprotein; M.P., Mycoplasma pneumoniae; MP, methylprednisolone; MRI, magnetic resonance imaging; mRS, modified Rankin Scale; N/A, not applicable; NGS, next-generation sequencing; TB DNA, tubercle bacillus DNA; TBA, tissue-based assay (rat brain sections); WBC, white blood cell.

a Normal range: 2.8–4.0 mmol/l.

b Autoimmune encephalitis antibodies: anti-N-methyl-D-aspartate receptor (NMDAR), leucine-rich glioma-inactivated 1 (LGI1), contactin-associated protein 2 (Caspr2), gamma-aminobutyric acid receptors B (GABA_B_R), alpha-amino-3-hydroxy-5-methylisoxazole-4-propionic acid receptors (AMPAR), dipeptidyl-peptidase-like protein-6 (DPPX), delta/notch-like epidermal growth factor-related receptor (DNER), dopamine-2 receptor (D2R), metabotropic glutamate receptor 5 (mGluR5), glutamate decarboxylase 65 kDa isoform (GAD65), IgLON family member 5 (IgLON5), and glycine receptor alpha 1 (GlyRα1) antibodies.

c Thyroid-related antibodies: anti-thyrotropin receptor, thyroid peroxidase, and thyroglobulin antibodies.

d Autoantibodies tested for patient 1 serum: anti-Hu (anti-neuronal nuclear antibody type 1, ANNA1), Ri (ANNA2), PNMA family member 2 (Ma2), ANNA3, SRY-box transcription factor 1 (SOX1), double-strand DNA (dsDNA), Smith (Sm), U1 small nuclear RNP (U1-RNP), Zic family member 4 (Zic4), Yo (Purkinje cell cytoplasmic antibody type 1, PCA1), amphiphysin, CRMP5, PCA2, recoverin, Titin, and Tr (DNER) antibodies.

e Autoantibodies tested for patient 2 serum: anti-dsDNA, Jo-1 (histidyl-tRNA synthetase, HARS), Sjögren’s syndrome-related antigen A (SSA/Ro52), SSB, Sm, U1-RNP, proliferating cell nuclear antigen, M2 type of antimitochondrial antibodies, centromere protein B, polymyositis scleroderma, Scl-70, and nucleosome AnuA antibodies.

f Tumor antigens/biomarkers: carbohydrate antigen (CA)-125, CA-199, CA-242, CA-724, cytokeratin-19 fragment (CYFRA21-1), squamous cell carcinoma antigen, carcinoembryonic antigen, and neuron-specific enolase.

### Study Design and Participants

P1’s CSF and/or serum samples were screened for known AE auto-Abs, including anti-NMDAR, LGI1, Caspr2, GABA_B_R, AMPAR, DPPX, DNER, D2R, mGluR5, GAD65, GlyRα1, and IgLON5 Abs by cell-based assay (CBA). It was conducted as immunofluorescence with antigen overexpressing HEK293T cells by transfection with corresponding plasmids, which contain the full-length human cDNAs of each antigen fused with GFP in vector pcDNA3.1-C-eGFP. Anti-Hu, Yo, Ri, Ma2, PCA2, amphiphysin, and CRMP5 Abs were tested with blot assay by Nanfang Precision Ltd. (Guangzhou, China). All the known auto-Abs tested were negative. The samples were further analyzed by TBA of immunostaining on rat brain sections and showed strong positive immunoreactive signals in the cytoplasm of neurons. It was considered with unknown Abs and applied for further Ab identification.

**Figure 2:**
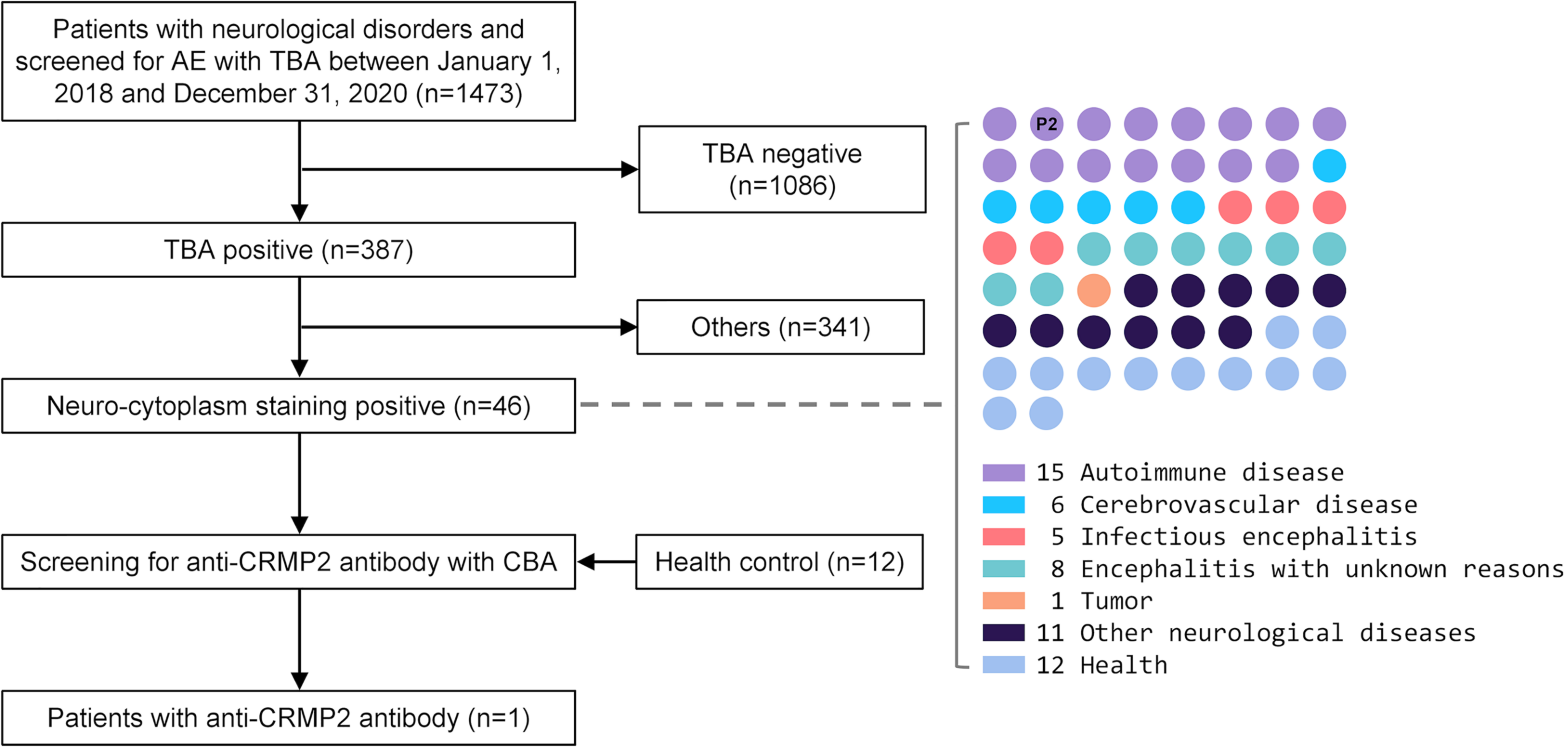
The flow chart of patients selected for anti-CRMP2 antibody screening. CSF and/or serum samples from 46 patients with neurological disorders that had positive immunostaining in the neuro-cytoplasm of rat brain sections were enrolled for retrospective anti-CRMP2 antibody screening. Twelve serum samples from healthy people were used as negative controls. A 42-year-old female patient with encephalomyelitis, right dot panel P2, was tested anti-CRMP2 antibody-positive in serum. AE, autoimmune encephalitis; CBA, cell-based assay; CRMP2, collapsin response mediator protein 2; CSF, cerebrospinal fluid; TBA, tissue-based assay.

For the retrospective study to find more anti-CRMP2 antibody-positive patients, we reviewed 1,473 patients admitted to our Neurology Department between January 1, 2018 and December 31, 2020 (**Figure 2**). These patients were cases of neurological disorders and had their TBA test performed. Forty-six patients with neuro-cytoplasm immunostainings in rat brain sections were finally enrolled for anti-CRMP2 antibody detection by CBA. Twelve serum samples from health people were used as controls.

### Immunohistochemistry

Frozen tissue sections, 5 μm in thickness, from the brain of adult SD rat or C57BL/6 mouse were prepared and performed by IHC (or TBA) with CSF, sera, and secondary Abs as previously reported (22). The dilutions used were as follows: CSF, 1:1; sera, 1:200; and DyLight 488-labeled goat anti-human IgG secondary Ab (ab97003, Abcam, Cambridge, MA), 1:200. Immunostaining images were photographed under an IX73 inverted microscope (Olympus, Tokyo, Japan) or LSM980 confocal microscope (Zeiss, Oberkochen, Germany). For the absorption assay, the serum with a dilution of 1:200 was pre-immunoabsorbed six times by incubation with acetone-fixed CRMP2-overexpressing HEK293T cells for 1 hour at 37°C as reported (6).

### Western Blotting, Immunoprecipitation, and Liquid Chromatography–Tandem Mass Spectrometry

Fresh whole-brain protein lysates of adult SD rats and Western blotting were processed as reported (23). A lysis buffer (20 mmol/l Tris-HCl, pH 7.4, 150 mmol/l, 1% TritonX-100, 2 mmol/l EDTA, 5% glyserol, 1 mmol/l PMSF) was used. The protein lysate was mixed with the patient’s CSF at 4°C overnight, followed by adding protein A agarose beads and incubating for another 2 h. The beads were washed, collected by centrifugation, and then boiled with SDS-PAGE sample buffer. The Ab–protein complexes pulled down were separated by 10% SDS gel electrophoresis, transferred to a nitrocellulose membrane for Western blotting, or stained with Coomassie brilliant blue. Correlated band compared with Western blotting results from the gels was cut and sent for protein identification using LC–MS/MS analysis by Fitgene Biotech Ltd. (Guangzhou, China).

### Cell Culture and Immunofluorescence

Primary cortical neurons were taken from 18- to 19-day-old C57BL/6 mouse fetuses and cultured for 14 days as reported (23). Ice acetone-fixed neurons were blocked with 10% goat serum at 37°C for 30 min and incubated with the patient’s serum (1:200), rabbit anti-MAP2 Ab (1:200, 8707T, Cell Signaling Technology, Danvers, MA, USA), or rabbit anti-CRMP2 Ab (1:200, ab129082, Abcam) at 4°C overnight. DyLight 488-labeled goat anti-human IgG and Alexa Fluor 594-labeled goat anti-rabbit IgG (ab150092, Abcam) secondary Abs (1:200) were used, and fluorescence images were photographed under LSM980 confocal microscope (Zeiss).

The HEK293T cells were transfected with plasmids pcDNA3.1-CRMP1/2/3/4-Flag, pcDNA3.1-CRMP5-eGFP, pcDNA3.1-CRMP2 T1 (1-141 aa of isoform 1)/T2 (1-36 aa of isoform 2)-eGFP, and pcDNA3.1-CRMP2 T3 (142-677 aa of isoform 1)-Flag separately for 48 h. The protein coding sequences in the plasmids referred to mRNAs of CRMP1: NM_001014809.3, CRMP2 (isoform 1): NM_001197293.3, CRMP2 (isoform 2): NM_001386.6, CRMP2 (isoform 3): NM_001244604.2, CRMP3: NM_006426.3, CRMP4: NM_001197294.2, and CRMP5: NM_020134.4. The full-length isoform 1 of CRMP2 was used unless specified otherwise. The transfected cells were fixed with ice acetone for 10 min, washed with PBST, blocked with 10% goat serum, and then incubated with the patient’s serum or rabbit anti-CRMP2 Ab (1:200) overnight at 4°C. After washing, the appropriate secondary Abs were incubated at room temperature for 1 h, including DyLight 488-labeled goat anti-human IgG, DyLight 550-labeled goat anti-human IgG (ab96908, Abcam), FITC-labeled anti-human IgG1/2/3/4 (F0767/F4516/F4641/F9890, Sigma-Aldrich), and/or Alexa Fluor 594-labeled goat anti-rabbit IgG secondary Abs (1:200). The fluorescence images were taken by using LSM980 confocal microscope (Zeiss) or IX73 inverted microscope (Olympus).

## Results

### Identification of an Auto-Ab Specifically Targeting CRMP2 in a Patient Suspected of AE

**Figure 3:**
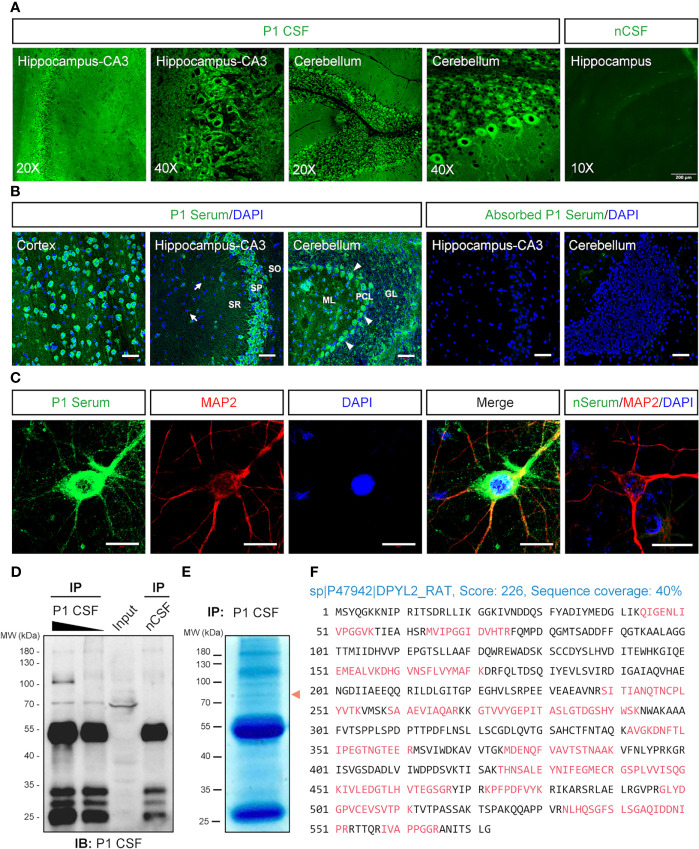
Identification of the autoantibody in a patient with acute encephalitis. P1 patient was diagnosed with encephalitis. The CSF sample from a control patient with carpal tunnel syndrome (nCSF) and the serum from a healthy person (nSerum) was used as negative controls. **(A)** Immunostaining on rat brain sections. The CSF from P1 patient had stained on hippocampus and cerebellum. **(B)** P1 patient’s serum stained obviously in the cytosolic compartments of the neurons in the cortex, CA3 area of the hippocampus, and cerebellum on mouse brain sections (green). White arrows, possible oligodendrocytes; arrow heads, Purkinje cells. CRMP2-expressing HEK293T cell absorbed P1 patient’s serum had no obvious staining. Cell nuclei were stained blue with DAPI. **(C)** P1 patient’s serum (green) stained positively in the soma and dendrites of cultured mouse cortical neurons which were labeled with anti-MAP2 Ab (red). **(D, E)** IP of P1 CSF with rat brain protein lysate. In total, 500 μl (lane 1) and 100 μl (lane 2) P1 CSF were applied to IP with 10 mg total protein of fresh rat brain lysate, respectively. Moreover, 500 μl nCSF was used for negative control. A positive band around 70 kDa was pulled down by P1 CSF but not by nCSF. A similar protein size band (red arrowhead) was observed on a parallel gel with Coomassie brilliant blue staining **(E)**. **(F)** Characterization of the autoantigen by mass spectrometry. The suspected protein band in **(E)** was collected for protein identification by LC-MS/MS. Seventeen identified peptides (red) matched rat DPYL2 (UniProtKB ID: P47942), an alias of CRMP2, with a score of 226 and sequence coverage of 40%. CRMP2, collapsin response mediator protein 2; CSF, cerebrospinal fluid; DAPI, 6-diamidino-2-phenylindole; DPYL2, dihydropyrimidinase like 2; GL, granular layer; IB, immunoblotting; IHC, immunohistochemistry; IP, immunoprecipitation; LC-MS/MS, liquid chromatography tandem mass spectrometry; MAP2, microtubule-associated protein 2; ML, molecular layer; PCL, Purkinje cell layer; SO, stratum oriens; SP, stratum pyramidale; SR, stratum radiatum. Scale bars represent 50 μm in **(B)** and 20 μm in **(C)**.

The serum and CSF samples were collected from a patient suspected of AE (P1). The patient was negative with the known auto-Abs, including anti-NMDAR, LGI1, Caspr2, GABA_B_R, AMPAR, DPPX, DNER, D2R, mGluR5, GAD65, and IgLON5 Abs by CBA and anti-Hu, Yo, Ri, Ma2, PCA2, amphiphysin, and CRMP5 by blotting assay. However, the immunofluorescence assay with P1’s CSF (**Figure 3A**) and serum (**Figure 3B**) revealed neuronal staining in the cortex, hippocampus, and especially the Purkinje cells of the cerebellum. A further analysis of P1’s serum showed immunostains in the cytosol compartments of cultured cortical neurons (**Figure 3C**).

IP complex pulled down from rat brain protein lysate by P1’s CSF was blotted with P1’s CSF, which revealed a band above 70 kDa (**Figure 3D**). The correlated visible band from the Coomassie brilliant blue staining gel (**Figure 3E**) was elucidated, and CRMP2 was identified as the possible target antigen by mass spectrometry (**Figure 3F** and **Supplementary Table S1**).

### Verification of the Anti-CRMP2 Ab and Screening in More Patients With Neurological Disorders

**Figure 4:**
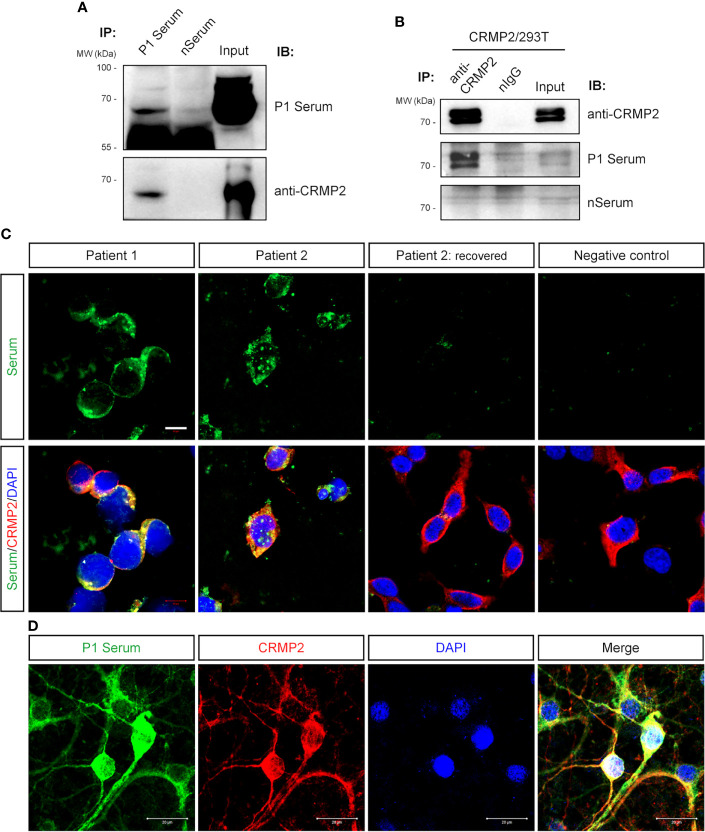
Verification of anti-CRMP2 antibody. **(A)** IP with P1 patient’s serum was performed with rat brain protein lysate. Immunoblottings were done with P1 patient’s serum (upper panel) and commercial anti-CRMP2 antibody (lower panel). **(B)** IP with commercial anti-CRMP2 antibody was performed with HEK293T cells overexpressing full-length human CRMP2 (isoform 1). Anti-CRMP2 antibody, P1 patient, or negative control sera were used for Western blotting, separately. **(C)** CRMP2-expressing HEK293T cells were immunostained with the patients’ sera and anti-CRMP2 antibodies. The sera from the first collection of P1 and P2 patients showed positive staining and co-localized with CRMP2. Serum collected from P2 patient 1.5 years after recovery showed negative staining. **(D)** Colocalization of P1 patient’s serum and anti-CRMP2 Ab immunostaining in cultured mouse cortical neurons. CRMP2, collapsin response mediator protein 2; DAPI, 6-diamidino-2-phenylindole; IB, immunoblotting; nIgG, normal immunoglobulin G; IP, immunoprecipitation; WB, Western blotting. Scale bars represent 10 μm in **(C)** and 20 μm in **(D)**.

Next, we confirmed that P1’s serum and commercial anti-CRMP2 Ab detected a band near 70 kDa from the pulled-down protein complex by P1’s serum from rat brain protein lysate (**Figure 4A**). With the CRMP2-overexpressing HEK293T cells, CRMP2 was pulled down as two bands above 70 kDa by commercial anti-CRMP2 Ab and recognized by P1’s serum (**Figure 4B**). There are three isoforms of CRMP2, isoform 1 (677 aa, 74 kDa), isoform 2 (572 aa, 62 kDa), and isoform 3 (536 aa, 58 kDa), which are attributed to three transcript variants. Here we used the human full-length isoform 1 CRMP2 for further testing. The observed CRMP2 bands with variable molecular size by Western blotting of different samples in **Figure 3D** and **Figures 4A, B** might be due to post-translational modification.

Anti-CRMP2 Ab and P1’s serum immunostained the overexpressed CRMP2 in HEK293T cells and were well co-localized (**Figure 4C**). As expected, immunostaining of cultured neurons with P1’s serum was also co-localized with anti-CRMP2 Ab signals (**Figure 4D**). When P1’s serum was pre-immunoabsorbed with the CRMP2-overexpressing HEK293T cells to rule out anti-CRMP2 Ab, a negative immunoreaction was found on the mouse brain section. These data indicated that no other neuronal Abs presented (**Figure 1B**).

Furthermore, we retrospectively screened for anti-CRMP2 Abs in 46 samples from other cases with neuro-cytoplasm immunostaining on rat brain sections, which included 15 autoimmune diseases, 6 cerebrovascular diseases, 5 infectious encephalitis, 8 encephalitis with unclear causes, 1 anaplastic astrocytoma, and 11 other neurological disorders (**Figure 2** and **Supplementary Table S2**). As indicated in **Figure 4C**, the serum of another patient, P2, with encephalomyelitis, had positive immunostains with CRMP2-overexpressing cells. Moreover, the positive stains from P2’s serum disappeared when the patient fully recovered and was reevaluated 1.5 years later.

### The Antibody Is IgG4 and Specifically Targets the C-Terminus of CRMP2

**Figure 5:**
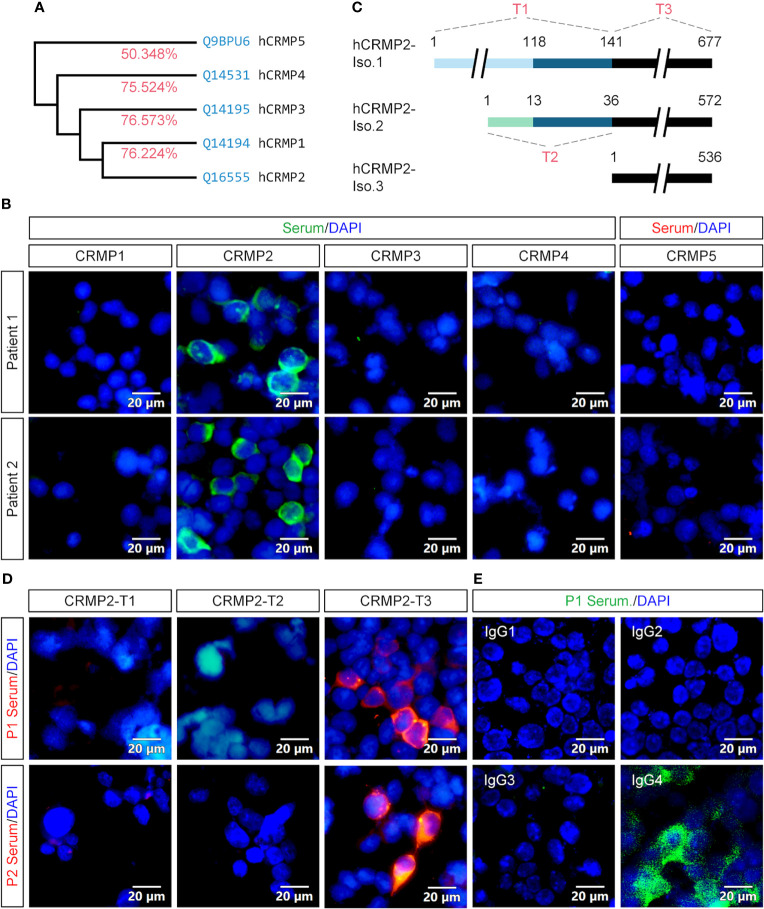
Specificity, epitope, and IgG subtype determination of the antibody. **(A)** Homology analysis showed that CRMP2 had an identity (red) of 76.224, 76.573, 75.524, and 50.348% with CRMP1, CRMP3, CRMP4, and CRMP5, respectively. Blue, UniProtKB accession number. **(B)** CBA assay of the patients’ sera for CRMPs. pcDNA3.1-based plasmids carrying CRMP1, CRMP2, CRMP3, CRMP4, and CRMP5-eGFP were transfected into HEK293T cells separately for protein overexpression. Secondary antibody anti-human IgG was labeled with green (CRMP1–4) or red (CRMP5) fluorescence. Cell nuclei were stained blue with DAPI. P1 (upper panels) and P2 (lower panels) patients’ sera were only immunoreactive with CRMP2 among CRMP family members. **(C)** Schematic drawings of the three CRMP2 isoforms and of the truncation strategy. The length or amino acid sites were indicated with numbers. Identical sequences between the isoforms were illustrated with the same line colors. **(D)** The pcDNA3.1-based plasmids carrying CRMP2 truncates T1, T2, or T3 illustrated in **(C)** were transfected into HEK293T cells separately for the CBA assay. P1 (upper panels) and P2 (lower panels) patient’s sera were reactive with T3 or 536 amino acid C-terminus of CRMP2 (red). **(E)** Immunostaining of CRMP2-expressing HEK293T cells with P1 patient serum and FITC conjugated anti-human IgG1-4 secondary antibodies. Positively stained in IgG4. CRMP, collapsin response mediator protein; DAPI, 6-diamidino-2-phenylindole; Iso., isoform. Scale bars represent 20 μm in **(B)**, **(D)**, and **(E)**.

Since CRMP2 belongs to the highly conserved CRMP family that currently has five members (**Figure 5A**), we wanted to further determine whether the Abs in our patients are specific to CRMP2. Expression plasmids were constructed to express full-length CRMP1–5. P1’s and P2’s sera showed immunostains only in the HEK293T cell expression of CRMP2, rather than other CRMPs (**Figure 5B**).

To further determine which part of CRMP2 was responsible for the antigen–antibody binding, three expression plasmids (T1–T3) carrying truncated CRMP2 were constructed as schematically indicated and applied to the CBA assay (**Figure 5C**). T1 contained 141 aa N-terminus of isoform 1, T2 had 36 aa N-terminus of isoform 2, and T3 had 536 aa C-terminus shared by three CRMP2 isoforms. The results indicate that the 536 aa C-terminus of CRMP2 contained the antigen epitope (**Figure 5D**).

We also checked the IgG subtype of anti-CRMP2 Ab in the two patients. As shown in **Figure 5E**, only IgG4 mediated the anti-CRMP2 immunoreaction. Identical results were obtained with P2’s serum (data not shown). These results suggested that anti-CRMP2 IgG4 recognized the C-terminus of CRMP2 in the two AE patients.

### Clinical Features of P2 Patient With Anti-CRMP2 Antibody

P2, a 42-year-old female patient, previously healthy, was admitted due to chest pain, right arm fatigue lasting for 10 days, headache, nausea, vomiting, and numbness in both legs lasting for 6 days. At 3 days before admission, the patient visited the emergency department of our hospital. The chest CT and electrocardiogram (ECG) were not remarkable, while the spine MRI showed long-segment spinal cord lesions from the medulla to the C6 segment (**Figures 1P–R**). On admission, the physical examination was not remarkable except for hyperalgesia in the left chest and abdomen, decreased needle tingling on the back of the left hand, and numbness in the feet. Lumbar puncture revealed an opening pressure of 185 mmH_2_O with moderately elevated WBC of 240 cells/µl (90% monocytes), decreased glucose of 2.64 mmol/l, elevated protein level of 0.80 g/l, and normal chloride level of 123 mmol/l. The CSF pathogen screenings for tuberculosis (TB) culture, TB-SPOT, X-pert, bacteria, and fungi cultures were all negative. Abs including AE panels, anti-aquaporin 4 (AQP4), anti-myelin oligodendrocyte glycoprotein (MOG) all resulted negative. Brain MRI with contrast revealed multiple patchy abnormal signals in the white matter area of the bilateral cerebral hemispheres and brain stem (**Figures 1M–O**). TBA was negative for CSF and positive for serum.

Possible tuberculous encephalomyelitis and acute demyelinating encephalomyelitis were considered. The patient was given an anti-TB regimen (rifampicin 0.45 g qd, isoniazid 0.3 g qd, pyrazinamide 0.5 g tid, and ethambutol 0.75 g qd) and intravenous MP pulse therapy (1 g/day for 5 days and tapered to oral 48 mg/day). Following the treatment, the patient’s chest pain and numbness gradually improved. A lumbar puncture was repeated 20 days later, which revealed that the WBC dramatically decreased to 20 cells/µl, while the glucose and protein returned to normal. The patient was followed up regularly in our clinic, and the steroids were tapered. The anti-TB therapy was discontinued after 6 months of treatment. A repeated brain MRI revealed the dramatic decrease of the abnormal signals in the white matter area of the bilateral cerebral hemispheres and brain stem (**Figures 1S–U**). A cervical MRI revealed that the abnormal signals in the spinal cord disappeared (**Figures 1V–X**).

## Discussion

In this study, we identified IgG4 auto-Ab specifically against CRMP2 in the sera and CSF samples of two patients with encephalitis or myelitis. The P1 patient dominantly presented signs of cerebellar encephalitis, including dizziness, slurred speech, and instability while walking. The P2 patient had encephalomyelitis, with the major complaint indicative of myelitis being chest pain and limb numbness. There was not enough evidence to support *M.P.* encephalitis in P1 or a possible TB in P2. However, TBA immunostaining strongly indicated a possible AE. Indeed immunotherapy led to a good improvement in these two patients at discharge, who achieved 2 and 0 score of mRS at 3 months (for P1 and P2, respectively). Currently, these patients are free of neoplasms, and they will be continuously followed up in the future. Therefore, the etiology of encephalitis remains unclear.

CRMPs are highly expressed during embryonic development and significantly decrease after birth, while each CRMP has a different subcellular localization (15). In the brain of an adult mouse, CRMP2 is expressed in the soma, axons, and dendrites of neurons from the cortex, hippocampus, cerebellum, and immature and mature oligodendrocytes (24, 25). It is thought that Abs against intracellular antigen mediate T cell immunoreaction and impair cell function in the related area, while the Abs themselves do not cause a pathogenic antibody–antigen interaction (26–28). Therefore, the symptoms of our two patients were consistent with the subcellular distribution of CRMP2 in the brain or probably in the spinal region. Indeed the sera/and CSF from the two patients with anti-CRMP2 Ab had robust staining of the cortex, CA3 region of the hippocampus, and Purkinje cells of the cerebellum.

Each CRMP has a distinct neuronal function. In mice with *Crmp2* gene knockout, it was found that lack of CRMP2 leads to defects in axon guidance, axon pruning in the hippocampus and visual cortex, and altered dendritic spine remodeling (29). The C-terminal region of CRMP2 is involved in post-translational modifications, including phosphorylation, SUMOylation, oxidation, and O-GlcNAcylation. Several critical phosphorylation sites are located in the C-terminus (30–32). The phosphorylated CRMP2 inhibits its interaction with tubulin heterodimers and augments the interaction with NaV1.7 (33). CRMP2 binds to CaV2.2 and increases the Ca^2+^ level on the cell surface. It also interacts with several receptors, including NMDARs, kainate, and NCX (Na^+^/Ca^2+^ exchanger 3) to regulate Ca^2+^ and Na^+^ neuronal homeostasis (34–36). Although immunoreaction of anti-CRMP2 in related neurons does not present a direct causative reason, it might lead to dysregulation of other receptors. Anti-ganglioside Abs inhibits peripheral axon regeneration *via* RhoA/Rock-dependent phosphorylation of CRMP2 at Thr555 (37). Epitope studies of CRMP2 have indicated that C-terminus is responsive to Ab binding, thus implying that an antigen–antibody reaction may affect the function of CRMP2. More studies should clarify the pathogenic properties of CRMP2 Ab.

*M.P.* encephalitis is one of the most severe extra-respiratory complications, accounting for 5–30% of all reported encephalitis cases (38). The latency period between the onset of respiratory symptoms and the development of neurological abnormalities varies. Some previous cases were without a previous clinically evident respiratory episode (39). P1 had a suspected intracranial infection of *M.P.* and early onset of encephalitis as evidenced by the presence of IgM antibody against mycoplasma in serum and the presence of *M.P.* DNA in CSF. The presence of antibodies to CRMP2 suggests that P1 had an immediately immune-mediated CNS inflammation, which was further supported by the fact that the sole treatment with antibiotics deteriorated the patient’s symptoms, yet additional IVIG and MP treatment gradually improved the symptoms. We considered that the symptoms of the patient were more due to the secondary inflammation caused by the *M.P.* infection. A similar study reports that mycoplasma infection led to secondary immune-mediated disease (40). Anti-ganglioside and galactocerebroside antibodies have been associated with mycoplasma neurological disorders. Thus, all these studies provide direct evidence that infection of *M.P.* may cause secondary immune-mediated inflammation (41).

We cannot exclude whether P2 could, in fact, have had tuberculosis based on the clinical features, CSF result, and improvement of treatment. The clinical improvement might be attributable to anti-tuberculosis treatment or steroids. Infections by viral and bacterial pathogens are suspected of initiating a broad range of neurological inflammatory disorders, particularly AE (42). Herpes simplex virus (HSV) has been reported to trigger some types of AE frequently more than others, including anti-NMDAR, GABA_A_R, mGluR5, Neurexin3a, and D2R antibodies. Among them, nearly 64% of AE patients who had post-HSV had anti-NMDAR antibodies in their serum or CSF, while the remaining cases had unidentified antibodies. While TB has been associated with numerous autoimmune disorders (43, 44), further studies are needed to establish whether TB is a trigger of anti-CRMP2 encephalitis in P2 patient.

In conclusion, our study characterized anti-CRMP2 Ab in two possible AE patients who responded well to immunotherapy. As the present study included small sample data from a single clinic center, future large cohort studies are needed to further verify the significance of anti-CRMP2 Ab associated with AE.

## Data Availability Statement

The original contributions presented in the study are included in the article/**Supplementary Material**. Further inquiries can be directed to the corresponding author/s.

## Ethics Statement

The studies involving human participants were reviewed and approved by the Ethical Review Committee of Nanfang Hospital, Southern Medical University, Guangzhou, China (permit number NFEC-2021-001). The patients/participants provided their written informed consent to participate in this study.

## Author Contributions

YHu, SP, YW, and KX designed the study and drafted the manuscript. DW and SW took care of the index patient and were responsible for the clinical data preparation. YHe and GL performed most of the experiments. YP provided cultured cells and neurons, HJ and YP were responsible for the other anti-CRMP2 Ab-positive patients. FX prepared the mouse and rat brain sections. YHuang took confocal images. QW performed Western blotting. All authors contributed to the article and approved the submitted version.

## Funding

This work was supported by the National Natural Science Foundation of China (81771225 to YHu, 82071484 to YW, and 82171602 to KX), Guangdong Provincial Scientific and Technologic Progression Fund (2016A020215182 to YHu), Natural Science Foundation of Guangdong Province (2019A1515011760 to YW), President Foundation of Nanfang Hospital (2019B007 to DW and 2020B008 to KX), and the Medical Science and Technology Foundation of Guangdong Province (A2021151 to KX).

## Conflict of Interest

A patent (ZL202110461611.6) is authorized and a PCT patent (PCT/CN2021/138864) is pending for the assay to detect the anti-CRMP2 Ab in autoimmune encephalitis or encephalomyelitis (SP, YHu, GL, SW, DW, KX, and YW).

The remaining authors declare that the research was conducted in the absence of any commercial or financial relationships that could be construed as a potential conflict of interest.

## Publisher’s Note

All claims expressed in this article are solely those of the authors and do not necessarily represent those of their affiliated organizations, or those of the publisher, the editors and the reviewers. Any product that may be evaluated in this article, or claim that may be made by its manufacturer, is not guaranteed or endorsed by the publisher.
