# Supplementary material for: Identification of Anti-Collapsin Response Mediator Protein 2 Antibodies in Patients With Encephalitis or Encephalomyelitis
Source: Front Immunol. 2022 Apr 11;13:854445. doi: 10.3389/fimmu.2022.854445 (PMC9036435; doi:10.3389/fimmu.2022.854445)
Supplement: Supplementary file 1 [file Table_1.docx]

**Supplementary tables**

**Table S1. CRMP2 peptides identified by LC-MS/MS after immunoprecipitation.**

| **Peptide sequence** | **Expected mass** | **Calculated mass** | **Mass deviation** | **Score** | **Expected score^b^** |
| --- | --- | --- | --- | --- | --- |
| R.IVAPPGGR.A^a^ | 765.4508 | 765.4497 | 0.0011 | 13.96 | 0.44 |
| K.SAAEVIAQAR.K | 1014.547 | 1014.5458 | 0.0013 | 38.75 | 0.0036 |
| K.SAAEVIAQAR.K | 1014.5472 | 1014.5458 | 0.0014 | 26.69 | 0.057 |
| R.GSPLVVISQGK.I | 1083.6296 | 1083.6288 | 0.0008 | 7.92 | 2.4 |
| R.GSPLVVISQGK.I | 1083.6307 | 1083.6288 | 0.0019 | 24.04 | 0.059 |
| R.KPFPDFVYK.R | 1139.602 | 1139.6015 | 0.0004 | 19.21 | 0.24 |
| R.KPFPDFVYK.R | 1139.6021 | 1139.6015 | 0.0006 | 25.56 | 0.057 |
| R.KPFPDFVYK.R | 1139.6027 | 1139.6015 | 0.0012 | 17.21 | 0.37 |
| R.KPFPDFVYK.R | 1139.6034 | 1139.6015 | 0.0018 | 7.51 | 3.5 |
| R.KPFPDFVYK.R | 1139.6034 | 1139.6015 | 0.0019 | 26.98 | 0.039 |
| K.GIQEEMEALVK.D | 1245.6292 | 1245.6275 | 0.0018 | 0.98 | 24 |
| K.GIQEEMEALVK.D | 1261.6219 | 1261.6224 | -0.0004 | 2.31 | 15 |
| R.MVIPGGIDVHTR.F | 1293.6879 | 1293.6864 | 0.0015 | 11.31 | 1.6 |
| K.QIGENLIVPGGVK.T^a^ | 1322.7553 | 1322.7558 | -0.0004 | 22.64 | 0.041 |
| K.QIGENLIVPGGVK.T^a^ | 1322.7561 | 1322.7558 | 0.0003 | 18.6 | 0.11 |
| R.GLYDGPVCEVSVTPK.T | 1619.7861 | 1619.7865 | -0.0004 | 10.8 | 1.9 |
| K.DHGVNSFLVYMAFK.D | 1642.7813 | 1642.7814 | 0 | 4.96 | 5.3 |
| K.DHGVNSFLVYMAFK.D | 1642.7827 | 1642.7814 | 0.0013 | 57.66 | 0.000029 |
| K.IVLEDGTLHVTEGSGR.Y | 1681.8663 | 1681.8635 | 0.0028 | 42.51 | 0.00094 |
| K.IVLEDGTLHVTEGSGR.Y | 1681.8673 | 1681.8635 | 0.0037 | 34.81 | 0.0056 |
| K.IVLEDGTLHVTEGSGR.Y | 1681.8698 | 1681.8635 | 0.0063 | 25.93 | 0.039 |
| K.MDENQFVAVTSTNAAK.V^a^ | 1740.7937 | 1740.7989 | -0.0052 | 6.17 | 3.4 |
| K.MDENQFVAVTSTNAAK.V^a^ | 1740.802 | 1740.7989 | 0.0031 | 1.09 | 12 |
| K.MDENQFVAVTSTNAAK.V^a^ | 1740.8092 | 1740.7989 | 0.0103 | 21.12 | 0.13 |
| K.DNFTLIPEGTNGTEER.M | 1791.83 | 1791.8275 | 0.0025 | 42.62 | 0.00098 |
| R.SITIANQTNCPLYVTK.V | 1821.9279 | 1821.9295 | -0.0015 | 46.93 | 0.0004 |
| K.THNSALEYNIFEGMECR.G | 2085.8886 | 2085.8884 | 0.0002 | 37.54 | 0.00085 |
| K.AVGKDNFTLIPEGTNGTEER.M | 2147.0524 | 2147.0495 | 0.003 | 8.27 | 2.9 |
| R.NLHQSGFSLSGAQIDDNIPR.R | 2168.067 | 2168.061 | 0.006 | 56.88 | 0.000035 |
| K.GTVVYGEPITASLGTDGSHYWSK.N | 2424.1666 | 2424.1598 | 0.0068 | 14.44 | 0.61 |

Abbreviations: CRMP2, collapsin response mediator protein 2; LC-MS/MS, liquid chromatography tandem mass spectrometry

^a^ Not unique peptides for CRMP2.

^b^ Peptides with expected score≤0.05 were considered significant.

**Table S2. Clinical information of patients screened for the presence of anti-CRMP2 antibody.**

| **Type** | **Number of patients** | **Diseases** | **Gender** | **Age** | **anti-CRMP2 Ab** |
| --- | --- | --- | --- | --- | --- |
| Autoimmune disease | 15 | Acute myelitis | Male | 22 | - |
|  |  | Encephalomyelitis^a^ | Female | 42 | + |
|  |  | ADEM | Male | 22 | - |
|  |  | Myelitis, anti-AQP4 Ab (+) | Female | 21 | - |
|  |  | Peripheral neuropathy, anti-Caspr1 Ab (+) | Male | 44 | - |
|  |  | Clinically isolated syndrome | Female | 25 | - |
|  |  | Demyelination pseudotumor | Male | 22 | - |
|  |  | Demyelination pseudotumor | Female | 30 | - |
|  |  | Dermatomyositis | Female | 75 | - |
|  |  | Guillain-barre syndrome | Male | 64 | - |
|  |  | Guillain-barre syndrome (possible) | Male | 74 | - |
|  |  | Hashimoto's encephalopathy | Male | 47 | - |
|  |  | anti-NMDAR encephalitis | Female | 77 | - |
|  |  | anti-NMDAR encephalitis | Female | 35 | - |
|  |  | Systemic lupus erythematosus, anti-UI-nRNP Ab (+) | Female | 22 | - |
| Cerebrovascular disease | 6 | Basilar artery apex syndrome | Male | 45 | - |
|  |  | Cerebral infarction | Female | 49 | - |
|  |  | Cerebral infarction | Female | 53 | - |
|  |  | Cerebral infarction | Male | 44 | - |
|  |  | Cerebral infarction | Female | 77 | - |
|  |  | Transient ischemic attack | Female | 29 | - |
| Infectious encephalitis | 5 | Encephalitis, Escherichia coli (+) | Female | 60 | - |
|  |  | Encephalitis, influenza A (H3N2) (+) | Female | 37 | - |
|  |  | Intracranial abscess, epilepsy | Male | 14 | - |
|  |  | Meningitis, Orientia tsutsugamushi (+) | Male | 53 | - |
|  |  | Encephalitis, status epilepticus, HSV1 (+) | Male | 53 | - |
| Encephalitis with unknown reasons | 8 | Possible tuberculous meningitis, anti-ANA, SSA, Ro52 Ab(+) | Female | 17 | - |
|  |  | Limbic encephalitis, EB virus (+) | Male | 52 | - |
|  |  | Epilepsy | Male | 20 | - |
|  |  | Encephalitis with unknown reasons | Female | 18 | - |
|  |  | Possible tuberculous meningitis | Male | 28 | - |
|  |  | Intracranial infection with unknown pathogen | Male | 53 | - |
|  |  | Intracranial infection with unknown pathogen | Male | 60 | - |
|  |  | New-onset refractory status epilepticus | Female | 27 | - |
| Tumor | 1 | Anaplastic astrocytoma | Male | 61 | - |
| Others | 11 | Fever & headache | Male | 58 | - |
|  |  | Multiple system atrophy | Male | 64 | - |
|  |  | Paralytic rabies | Male | 56 | - |
|  |  | Possible retrobulbar neuritis | Male | 52 | - |
|  |  | Possible mitochondrial encephalomyopathy | Female | 60 | - |
|  |  | Schizophrenia | Male | 16 | - |
|  |  | Anxiety | Female | 43 | - |
|  |  | Possible Amyotrophic Laternal Sclerosis | Male | 66 | - |
|  |  | Peripheral neuropathy (amyloidosis), ANA (+) | Male | 65 | - |
|  |  | Brain stem space occupying lesions | Female | 39 | - |
|  |  | Antineoplastic drug-associated encephalopathy | Male | 66 | - |
| Health | 12 | N/A | Female | 37 | - |
|  |  | N/A | Male | 68 | - |
|  |  | N/A | Female | 63 | - |
|  |  | N/A | Male | 19 | - |
|  |  | N/A | Male | 41 | - |
|  |  | N/A | Female | 43 | - |
|  |  | N/A | Male | 76 | - |
|  |  | N/A | Female | 74 | - |
|  |  | N/A | Female | 27 | - |
|  |  | N/A | Male | 54 | - |
|  |  | N/A | Male | 51 | - |
|  |  | N/A | Male | 48 | - |
| Total | 58 |  | Male/Female (33/25) | Mean±SD (45.8±18.5) | Positive (1/58, 1.7%) |

Abbreviations: Ab, antibodies; ADEM, acute demyelinating encephalomyelitis; ANA, antinuclear antibody; AQP4, aquaporin 4; Caspr1, contactin-associated protein 1; Epstein-Barr (EB) virus; HSV1, herpes simplex virus; N/A, not applicable; NMDAR, N-methyl-D-aspartate receptor; Sjögren's-syndrome-related antigen A (SSA/Ro52); U1-nRNP, U1 small nuclear RNP.

^a^ P2 patient in the present study.
